# Revision of the Cyclisation Mechanism for the Diterpene Spiroviolene and Investigations of Its Mass Spectrometric Fragmentation

**DOI:** 10.1002/cbic.202000682

**Published:** 2020-11-10

**Authors:** Houchao Xu, Jeroen S. Dickschat

**Affiliations:** ^1^ Kekulé Institute for Organic Chemistry and Biochemistry University of Bonn Gerhard-Domagk-Straße 1 53121 Bonn Germany

**Keywords:** biosynthesis, enzyme mechanisms, isotopes, mass spectrometry, terpenes

## Abstract

The diterpene spiroviolene, its diterpene synthase from *Streptomyces violens* and the experimentally determined terpene cyclisation mechanism were reported in 2017. Recently, the structure of spiroviolene was revised based on a total synthesis, with consequences for the cyclisation mechanism. Herein, a reinvestigation of the terpene cyclisation to spiroviolene and the mass spectrometric fragmentation mechanism investigated by ^13^C‐labelling experiments are presented.

## Graphical abstract

**Second thoughts**: After the structural revision of the bacterial diterpene spiroviolene its biosynthesis is revisited and can now be integrated into a unified scheme with related fungal compounds such as the cyclopiane‐type diterpenes and spirograterpene A. An isotopic labelling study giving insights into the EI‐MS fragmentation mechanism of spiroviolene is presented.

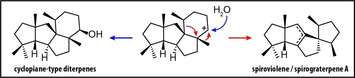

Diterpenes are made by diterpene synthases (DTSs) that convert the acyclic and achiral precursor geranylgeranyl diphosphate (GGPP) in remarkable one‐step enzymatic transformations into usually enantiomerically pure, often polycyclic, sometimes even cage‐like molecules with multiple stereogenic centres. Several type I DTSs were recently discovered from bacteria which are monofunctional enzymes,^[1]^ while fungal DTSs are usually bifunctional and exhibit a prenyltransferase (GGPP synthase, GGPPS) and a DTS domain.[^2^, ^3^] Spiroviolene is a spirocyclic triquinane diterpene from *Streptomyces violens* for which we had originally reported the structure of **1 a** (Scheme 1A). The compound is made by a diterpene synthase that has been deeply studied for its cyclisation mechanism through the use of several isotopically labelled substrates.^[4]^ In particular, a double labelling experiment with (3‐^13^C,2‐^2^H)GGPP resulted in an upfield shifted triplet for C3 in the ^13^C NMR spectrum, indicating a direct ^13^C,^2^H bond in labelled **1** obtained with spiroviolene synthase (SvS) from this substrate. This result was interpreted by the cyclisation mechanism shown in Scheme 1B in which the cationic intermediate **E** reacts by a 1,3‐hydride migration to **F**, establishing the stereogenic centre at C3. Notably, the structure of **1 a** differs with respect to the configuration of the stereogenic centre at this carbon from those of structurally similar molecules, including the fungal cyclopiane‐type diterpenes conidiogenol (**4**) and conidiogenone (**5**) from *Penicillium cyclopium*,^[5]^ several derivatives from other *Penicillium* spp.,[^6^, ^7^] and spirograterpene A (**2**) from *Penicillium granulatum* that has the same skeleton as **1**.^[8]^ Also the bifunctional cyclopiane‐type diterpene synthase from *Penicillium chrysogenum* has been reported that is responsible for the biosynthesis of **3** as the proposed precursor to **4**, **5** and other cyclopiane type diterpenes. In this study, the structure of compound **3** was established by X‐ray analysis.^[9]^ Recently, the structure of the bacterial compound **1 a** was revised by Snyder and co‐workers to that of **1 b** based on a total synthesis of both stereoisomers, showing that the stereogenic centre at C3 of **1** has the same configuration as for the fungal compounds.^[10]^ This finding has consequences on the cyclisation mechanism, because the proposed 1,3‐hydride shift from **E** to **F** can only proceed with the facial selectivity to explain the formation of **1 a**, but cannot explain the revised stereochemistry at C3 in **1 b**. Herein, we report additional labelling experiments that further support the revised structure of **1 b** for spiroviolene, a new biosynthetic hypothesis that is in line with all previous labelling experiments and explains the formation of **1 b**, and the EI‐MS fragmentation mechanism of spiroviolene based on ^13^C‐labelling of each individual carbon.

**Scheme 1:**
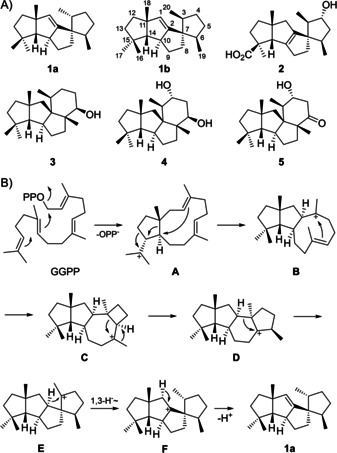
A) Originally reported (**1 a**) and revised (**1 b**) structures of spiroviolene from *S. violens* and related compounds from *Penicillium*. B) Originally proposed biosynthetic hypothesis for the cyclisation of GGPP to **1 a**.

During the original structure elucidation of **1**, the assignment of the relative configuration for its western half by interpretation of the NOESY spectrum proved to be clear, while the assignment for the relative configuration at C3 turned out to be difficult. This was also the case for the NOESY based assignment of the diastereotopic hydrogens H_α_ and H_β_ at some of the methylene carbons that was undoubtedly established for C5 and C9 by enantioselective deuterations using the substrates (*R*)‐ and (*S*)‐(1‐^13^C,1‐^2^H)farnesyl diphosphate (FPP) as well as (*R*)‐ and (*S*)‐(1‐^13^C,1‐^2^H)geranyl diphosphate (GPP) in conjunction with isopentenyl diphosphate (IPP) and GGPPS from *Streptomyces cyaneofuscatus*.^[4]^ Meanwhile, additional stereoselectively deuterated substrates have been made available by synthesis in our laboratory, including (*R*)‐ and (*S*)‐(1‐^13^C,1‐^2^H)IPP that can be used with isopentenyl diphosphate isomerase (IDI) and GGPPS to introduce enantioselective deuterations at C1, C5, C9, and C13 of GGPP.^[11]^ Furthermore, (*E*)‐ and (*Z*)‐(4‐^13^C,4‐^2^H)IPP together with dimethylallyl diphosphate (DMAPP) and GGPPS give rise to enantioselectively deuterated GGPP at C4, C8 and C12.^[12]^ The additional ^13^C‐labelling at the deuterated carbons allow for a sensitive detection of the connected hydrogens by HSQC spectroscopy, while the signal for the hydrogen replaced by deuterium is extinguished. Data interpretation in these experiments is based on the fundamental work by Cornforth and co‐workers on the stereochemical course of the prenyltransferase reaction.^[13]^

The enzymatic conversions of these probes with SvS and HSQC analysis of the obtained products (Table S1 and Figures S1–S4 in the Supporting Information) resulted in the assignments for the diastereotopic hydrogens as summarised in Scheme 2A, showing that the original assignments for the hydrogens at C4 and C12 require revision (Table 1). While the erroneous assignments for H4_α_ and H4_β_ fitted better to the structure of **1 a** with a pseudo‐*C*_2_ axis (ψ‐*C*_2_) for the eastern cyclopentane ring (Scheme 2B), the corrections for H4_α_ and H4_β_ are in line with Snyder's revised structure of **1 b** with a pseudo‐symmetry plane (ψ‐σ). This also demonstrates how useful the stereoselectively deuterated precursor probes are for the structure elucidation of terpenes.

**Scheme 2:**
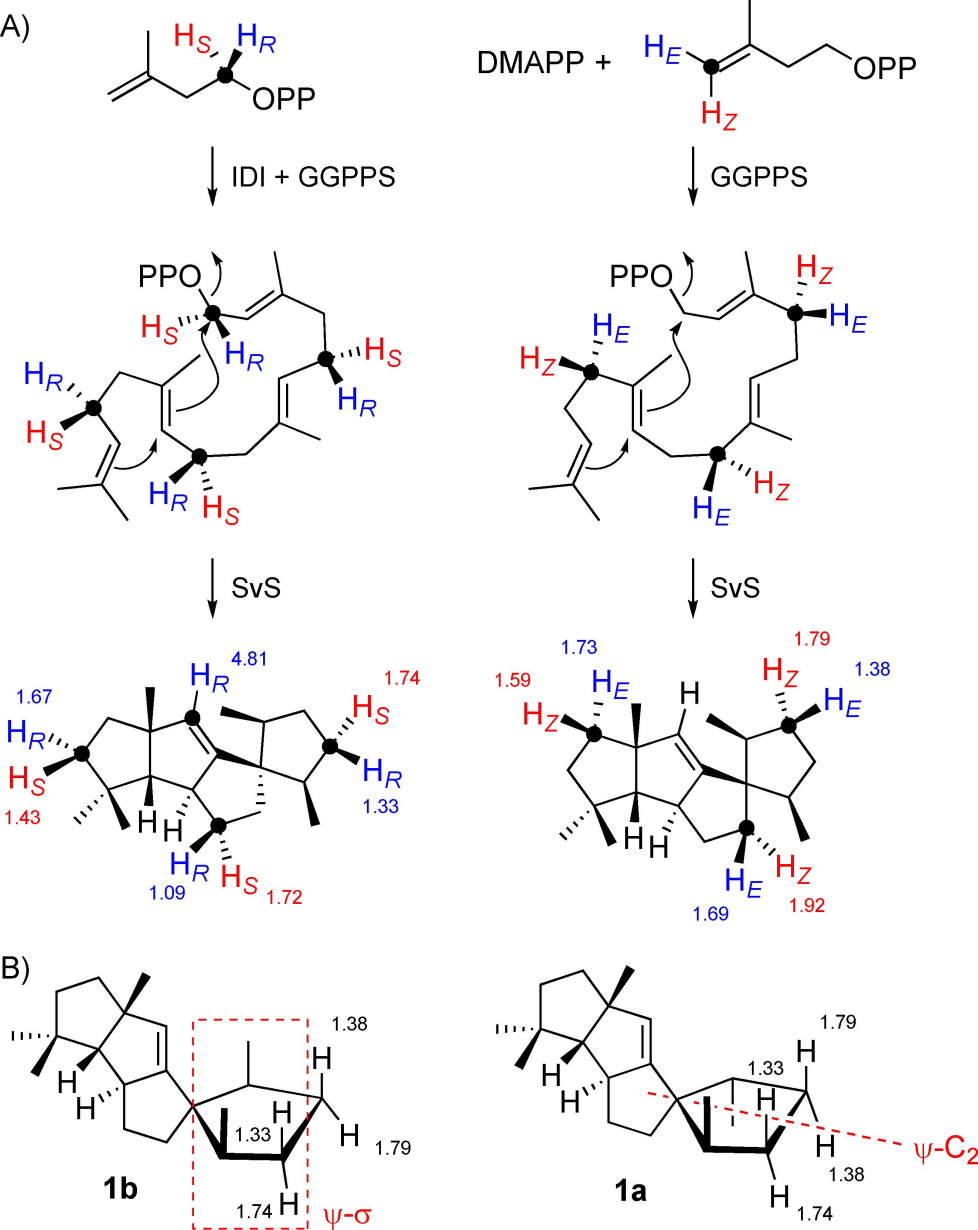
A) Enantioselective deuteration of **1 b** and resulting assignments of the NMR data for hydrogens at methylene groups. Data at hydrogens indicate ^1^H chemical shifts in ppm. B) Pseudo‐symmetry plane (ψ‐σ) in **1 b** with corrected NMR assignments for H4_α_ and H4_β_, and pseudo‐*C*_2_ axis (ψ‐*C*_2_) in the eastern cyclopentane ring of **1 a** with original erroneous assignments.

**Table 1:** Revised NMR data for spiroviolene (**1**) in C_6_D_6_ (700 MHz).

C^[a]^	type	^13^C^[b]^	^1^H^[b]^
1	CH	128.9	4.81 (d, *J*=2.9)
2	C_q_	148.9	–
3	CH	44.7	1.60 (m)
4	CH_2_	31.3	1.79 (m, H_α_)* 1.38 (m, H_β_)*
5	CH_2_	30.7	1.74 (m, H_α_) 1.33 (m, H_β_)
6	CH	46.6	1.81 (m)
7	C_q_	53.8	–
8	CH_2_	39.5	1.92 (ddd, *J*=12.7, 6.9, 6.9, H_α_) 1.69 (m, H_β_)
9	CH_2_	33.1	1.72 (m, H_α_) 1.09 (dddd, *J*=12.2, 12.2, 11.3, 7.6, H_β_)
10	CH	59.4	2.77 (dddd, *J*=12.5, 6.4, 6.4, 2.9)
11	C_q_	63.7	–
12	CH_2_	38.6	1.73 (m, H_α_)* 1.59 (m, H_β_)*
13	CH_2_	40.8	1.67 (m, H_α_) 1.43 (dddd, *J*=11.8, 6.6, 1.5, 1.5, H_β_)
14	CH	66.0	1.58 (m)
15	C_q_	41.3	–
16	CH_3_	29.1	1.04 (s)
17	CH_3_	26.1	1.03 (s)
18	CH_3_	32.4	1.34 (s)
19	CH_3_	15.2	0.97 (d, *J*=6.7)
20	CH_3_	15.1	0.94 (d, *J*=6.7)

[a] Carbon numbering as shown in Scheme 1. [b] Chemical shifts δ in ppm, multiplicity (s=singlet, d=doublet, m=multiplet), and coupling constants *J* in Hz.

After the structural revision of **1**, a modified biosynthetic proposal is required that can now be unified to a general biosynthetic hypothesis for bacterial spiroviolene and the fungal cyclopiane‐type diterpenes (Scheme 3). Following a 1,11–10,14 cyclisation of GGPP to **A**′ (=**A** in Scheme 1), the next two steps represent a modification of our earlier proposal and are similar to the initial steps in variediene biosynthesis suggested by Hong and Tantillo based on DFT calculations.^[14]^ This includes expansion of the cyclopentane ring, followed by a transformation that was described as “highly asynchronous ring‐opening/ring‐closing process that accomplishes the same net result as a 1,2‐alkyl shift“^[14]^ from C10 to C14, as indicated in **B**′. This reaction, together with a 2,10‐cyclisation, leads to **C**′ (=**B** in Scheme 1, note that the stereochemistry of **C’** at C2 is different to the corresponding intermediate proposed for variediene biosynthesis^[14]^). Cation **C**′ then reacts by a 1,2‐hydride shift from C2 to C3 to yield **D**′, which substitutes for the 1,3‐hydride migration that we had established experimentally in our previous study,^[4]^ but is now reinterpreted to explain the corrected stereochemistry at C3 of **1**.^[10]^ The following 2,7‐cyclisation then leads to the secondary cation **E’** that can be trapped by water to yield **3**,^[9]^ a compound that may be oxidised via **4** to **5**,^[5]^ for example, by cytochrome P450 oxygenases. The configuration of the stereocentre at the carbinol carbon in **3** is in line with a concerted **D**′‐to‐**3** conversion with *anti* addition to the double bond between C6 and C7 in **D**′. Alternatively, in the absence of water **E**′ may transiently react through the nonclassical cation **F’** to **G**′ with Me19 now being shifted from C7 to C6, which represents a skeletal rearrangement that was established experimentally in our previous study by ^13^C‐labelling of each individual carbon and substitutes for the **C**‐to‐**D** transformation in Scheme 1.^[4]^ The spirocentre is installed by ring contraction of **G**′ to **H**′, substituting for the ring contraction from **D** to **E** in Scheme 1. Cation **H**′ can react by alternative deprotonations to spiroviolene (**1**) or the hypothetical natural product **6** that likely serves as the precursor to spirograterpene A (**2**).^[8]^ Notably, in the final deprotonations to **1** and **6** the proton is abstracted from the same hemisphere of **H**′, for which the selectivity was supported by stereoselective deuteration at C1 for **1**.^[4]^

**Scheme 3:**
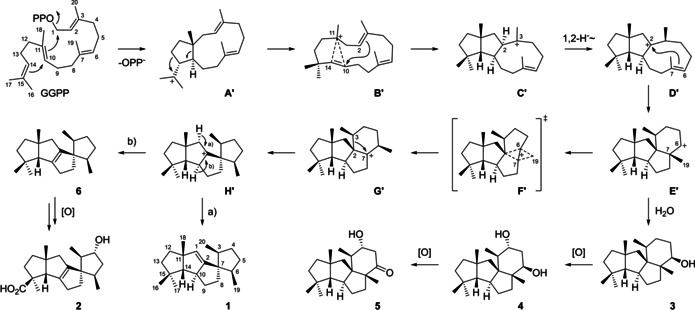
Revised cyclisation mechanism from GGPP to **1** and biosynthetic links to related fungal compounds.

Besides for biosynthetic investigations, isotopically labelled compounds are also very useful to study mass spectrometric fragmentation mechanisms (for important mass spectrometric fragmentation reactions such as σ‐bond cleavage, α‐fragmentation, inductive cleavage, McLafferty rearrangement and retro‐Diels‐Alder fragmentation, cf. ref. [15]). The required isotopic labelling can be introduced into well‐defined positions by feeding of correspondingly labelled precursors to cultures of the producing organisms, but if the incorporation rates are low, mixtures of different isotopomers and/or of the labelled and the unlabelled compound will be obtained, which can significantly hamper data interpretation. Synthetic or semisynthetic approaches can give access to compounds with well‐defined isotopic substitutions, but are very laborious and might require different synthetic strategies for each target position of the natural product. Deuterium has often been used successfully to study EI‐MS fragmentation mechanisms of terpenes,[^16^, ^17^, ^18^, ^19^, ^20^] but sometimes gave unclear results as a consequence of unspecific scrambling.^[21]^ In previous studies on EI‐MS fragmentation mechanisms we have enzymatically prepared ^13^C‐labelled terpenes from the corresponding synthetic ^13^C‐labelled terpene precursors, which allowed to label each individual carbon.[^22^, ^23^, ^24^, ^25^, ^26^] Using the same approach all 20 isotopomers of (^13^C)‐**1** were enzymatically prepared from labelled terpene precursors with SvS (Table S1). Based on a comparison of their mass spectra (Figure S5) to the mass spectrum of unlabelled **1** (Figure 1), a position‐specific mass shift analysis (PMA_*m*/*z*_) indicates for a studied fragment ion (*m*/*z*) which carbons contribute to its formation (Figure 2).

**Figure 1:**
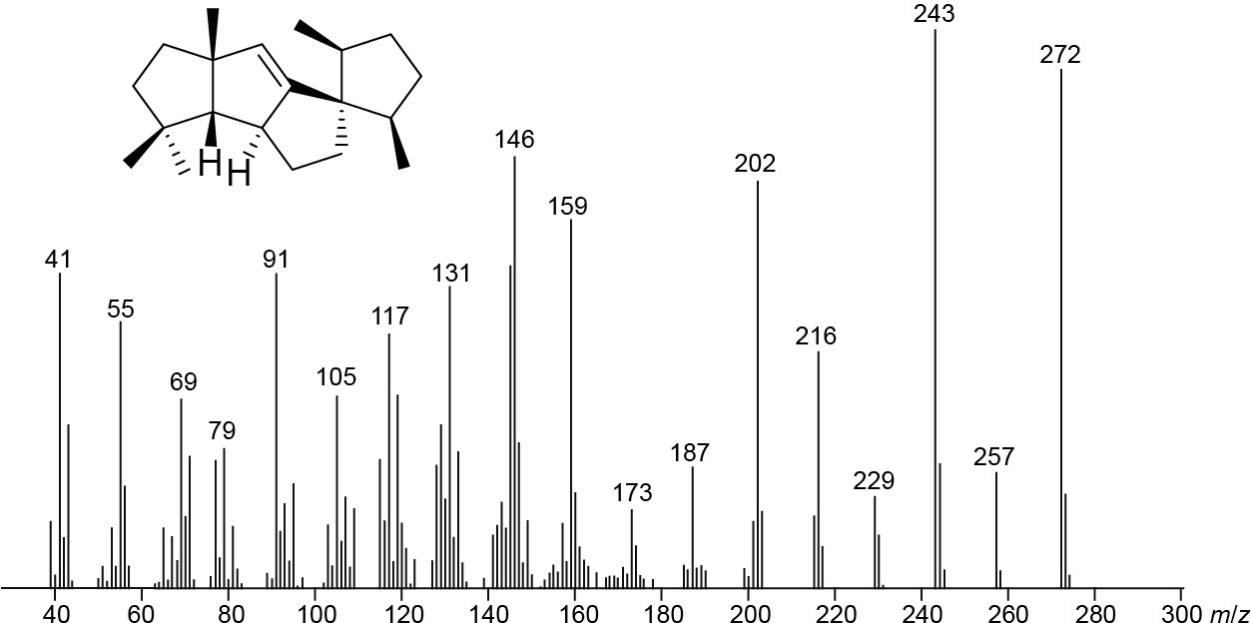
EI mass spectrum of **1**.

**Figure 2:**
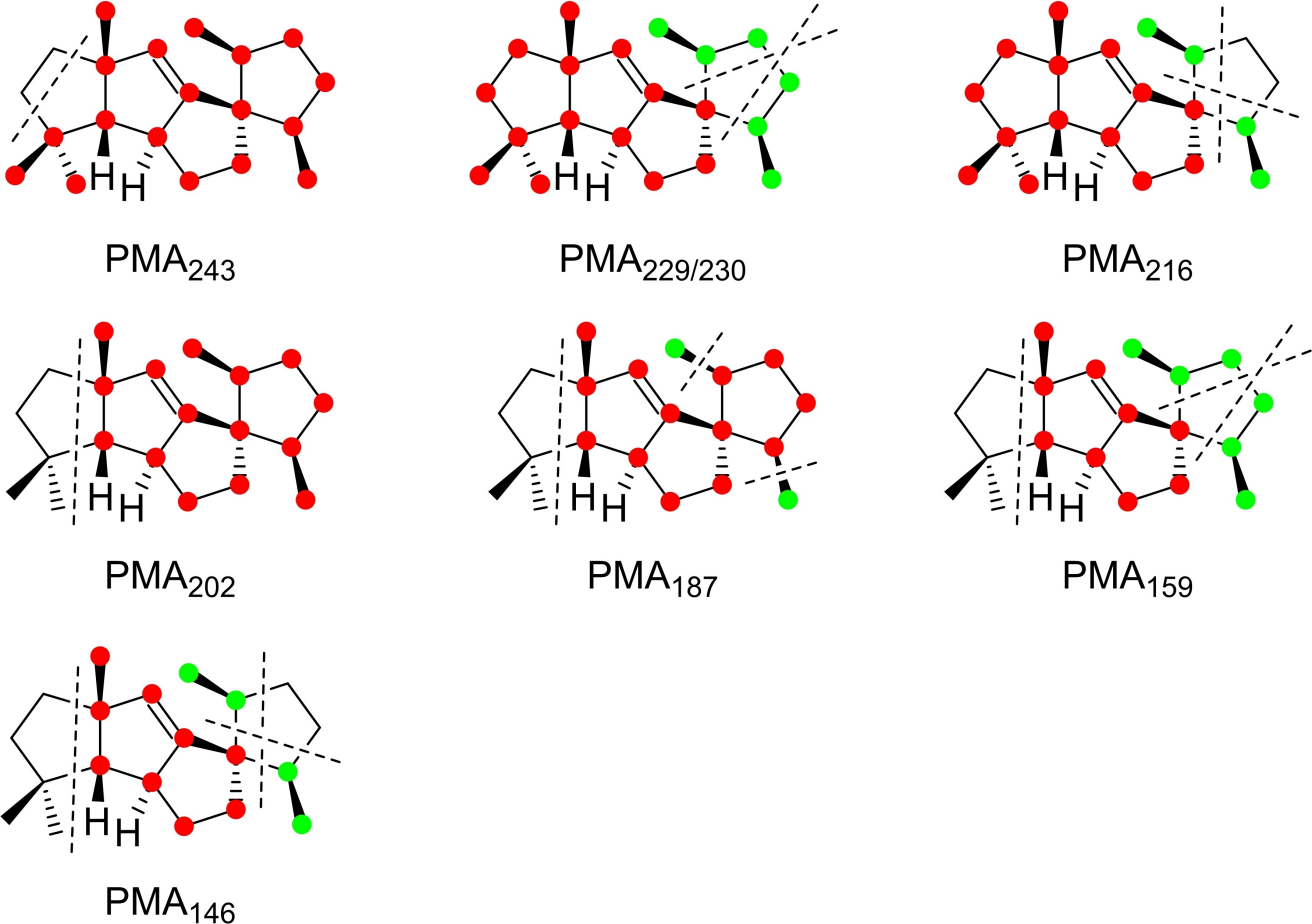
Position‐specific mass‐shift analysis (PMA_*m*/*z*_) for main EI fragment ions *m*/*z* of **1**. Red dots indicate carbons that contribute fully, green dots indicate carbons that contribute partially to the formation of a fragment ion. Dotted lines indicate relevant carbon–carbon bond cleavages.

The PMA for *m*/*z* 243 ([*M*−C_2_H_5_]^+^) reveals the specific formation of this fragment ion by cleavage of C12 and C13. After electron impact ionisation of **1** to the radical cation **1** ^.+^, this is explainable by a sequence of α‐cleavage to **a** ^.+^, hydrogen rearrangement to the conjugated butadienyl cation **b** ^.+^, and another α‐fragmentation to **c** ^+^ (Scheme 4A). The PMAs for *m*/*z* 230 ([*M*−C_3_H_6_]^+^) and 229 ([*M*−C_3_H_7_]^+^) demonstrate their formation from the same portion of **1**, with partial loss of three of the carbons from the C20‐C3‐C4‐C5‐C6‐C19 unit. Most likely, the lost carbons extrude as an intact portion C20‐C3‐C4 or C5‐C6‐C19. Starting from **1** ^.+^ two sequential α‐cleavages can proceed via **d** ^.+^ to **e** ^.+^, or by alternative hydrogen rearrangement of **d** ^.+^ to **f** ^.+^ and α‐fragmentation to the pentadienyl cation **g** ^+^. Both reactions are shown in Scheme 4B for the loss of the C20‐C3‐C4 unit, while analogous reactions can explain cleavage of carbons C5‐C6‐C19. The fragmentation mechanism towards *m*/*z* 216 ([*M*−C_4_H_8_]^+^) with cleavage of C4‐C5 and either C20‐C3 or C6‐C19 can similarly be understood by loss of an intact unit C20‐C3‐C4‐C5 or C4‐C5‐C6‐C19. Scheme 4C shows possible fragmentation reactions for the first case starting from **d** ^.+^ by hydrogen rearrangement to **h** ^.+^ and α‐fragmentation with loss of butene to **i** ^.+^. The PMA for *m*/*z* 202 ([*M*−C_5_H_10_]^+^) indicates the formation of this fragment ion by specific cleavage of C12‐C13‐C15(−C17)‐C16. This finding can be explained by hydrogen rearrangement from **a** ^.+^ to **j** ^.+^ and α‐fragmentation with fragmentation of 2‐methylbut‐2‐ene to **k** ^.+^ (Scheme 4D).

**Scheme 4:**
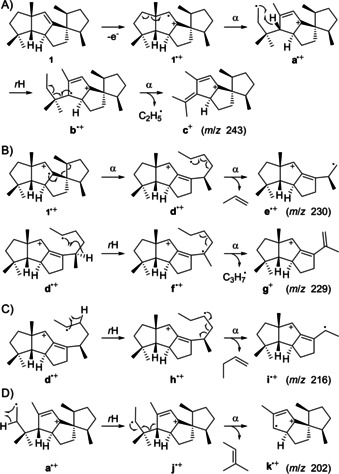
Fragmentation mechanisms for fragment ions *m*/*z* 243, 230, 229, 216 and 202 of **1**. α: α‐cleavage, *r*H: hydrogen rearrangement.

The PMAs for *m*/*z* 187, 159 and 146 (Figure 2) reveal that these fragments arise by a two‐step process with loss of the C12‐C13‐C15(−C17)‐C16 portion in all cases, in addition to cleavage of a methyl group (C19 or C20), or of a C_3_H_7_ or a C_4_H_8_ fragment as in the formation of **g** ^+^ or **h** ^.+^, respectively. Starting from **k** ^.+^, an α‐cleavage to **l** ^.+^, hydrogen rearrangement to **m** ^.+^, and another α‐fragmentation yield **n** ^+^ with a conjugated heptatrienyl cation system to explain *m*/*z* 187 [*M*−C_5_H_10_‐CH_3_]^+^ (Scheme 5A, shown for the loss of C19, the loss of C20 can proceed analogously). A similar sequence through **l** ^.+^, hydrogen rearrangement to **o** ^.+^, and α‐fragmentation gives **p** ^+^ to explain *m*/*z* 159, again hypothetically best represented by a conjugated heptatrienyl cation ([*M*−C_5_H_10_‐C_3_H_7_]^+^, Scheme 5B). The fragment ion at *m*/*z* 146 ([*M*−C_5_H_10_‐C_4_H_8_]^+^) can be formed from **l** ^.+^ by hydrogen rearrangement to **q** ^.+^ and subsequent α‐cleavage to the hexatrienyl cation **r** ^.+^ (Scheme 5C).

**Scheme 5:**
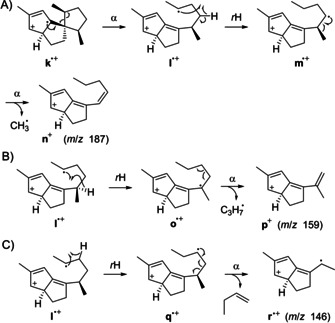
Fragmentation mechanisms for fragment ions *m*/*z* 187, 159 and 146 of **1**. α: α‐cleavage, *r*H: hydrogen rearrangement.

## Conclusion

The structural revision of spiroviolene (**1**) as recently promoted based on a total synthesis by Snyder and co‐workers^[10]^ has prompted us to reinvestigate the complete NMR data assignment through the use of stereoselectively deuterated precursors. As discussed here, the now rigorously assigned data are in line with and further support the structural revision of **1**. Moreover, a revised biosynthetic hypothesis for the terpene cyclisation of GGPP to **1** has been developed. The previously suggested biosynthetic hypothesis for the initially assigned structure of **1** was fully supported by extensive isotopic labelling experiments, as is the revised mechanism for the corrected structure of **1**, demonstrating that enzyme mechanistic models, like reaction mechanisms for any chemical reaction, can only be supported by experimental data, while absolute proof for chemical mechanistic models is in principle impossible to obtain. The revised structure of **1** now allows for a unified hypothesis of a biosynthetic mechanism towards this compound and several structurally related diterpenes from fungi. The DTC domain of the cyclopiane‐type diterpene synthase from *P. chrysogenum* ^[9]^ has only 15 % amino acid sequence identity to SvS,^[4]^ demonstrating that similar functions have evolved independently in fungi and bacteria. A similar finding was made previously for the fungal and bacterial diterpene synthases for phomopsene,[^27^, ^28^] while the fungal and bacterial sesquiterpene synthases for corvol ethers have a common evolutionary origin, suggesting cross‐kingdom horizontal gene transfer.[^29^, ^30^] Besides biosynthetic and enzyme mechanistic investigations, isotopically labelled terpene precursors can give valuable insights into EI‐MS fragmentation mechanisms of terpenes, as the enzymatic access of specifically labelled terpenes from these precursors, after their individual chemical synthesis, is straight forward and superior to a chemical synthesis of all positional singly ^13^C‐labelled isotopomers of terpenes.

## Conflict of interest

The authors declare no conflict of interest.

## Supporting information

As a service to our authors and readers, this journal provides supporting information supplied by the authors. Such materials are peer reviewed and may be re‐organized for online delivery, but are not copy‐edited or typeset. Technical support issues arising from supporting information (other than missing files) should be addressed to the authors.

Supplementary
