# Supplementary material for: Revision of the Cyclisation Mechanism for the Diterpene Spiroviolene and Investigations of Its Mass Spectrometric Fragmentation
Source: Chembiochem. 2020 Nov 10;22(5):850–4. doi: 10.1002/cbic.202000682 (PMC7983979; doi:10.1002/cbic.202000682)
Supplement: Supplementary file 1 — Supplementary [file CBIC-22-850-s001.pdf]

ChemBioChem

Supporting Information

Revision of the Cyclisation Mechanism for the Diterpene Spiroviolene and Investigations of Its Mass Spectrometric Fragmentation

Houchao Xu and Jeroen S. Dickschat*

Isotopic labelling experiments for reassignment of NMR data of 1

The isotopic labelling experiments (Table S1) with (*R*)- or (*S*)-(1-^{13}C,1-^{2}H)IPP, or DMAPP plus (*E*)- or (*Z*)-(4-^{13}C,4-^{2}H)IPP were performed in total volumes of 10 mL. Each substrate (1 mg) was dissolved in aqueous NH_4HCO_3 solution (1 mL, 25 mM). The required enzymes (SvS, IDI and GGPPS) were expressed in *E. coli* and purified as reported previously.[1,2] Enzyme elution fractions from Ni^{2+} NTA affinity chromatography (1 mL each) and incubation buffer (to a total volume of 10 mL) were added. After incubation with shaking at 28 °C overnight, the reaction mixtures were extracted with C_6D_6 (0.9 mL). The extracts were centrifuged to remove water and directly analysed by NMR and GC/MS.

Isotopic labelling experiments for EI-MS fragmentation mechanism of 1

The isotopic labelling experiments targeting the 20 carbons of 1 were performed with the substrates and enzymes as listed in Table S1 in total volumes of 1 mL, containing substrates (0.5 mg each) in aqueous NH_4HCO_3 solution (0.1 mL, 25 mM), enzyme elution fractions (0.2 mL each) and incubation buffer (0.5 mL). After incubation with shaking at 28 °C overnight, the reaction mixtures were extracted with n-hexane (0.2 mL). The extracts were centrifuged to remove water and directly analysed by GC/MS.

NMR spectroscopy

NMR data were recorded on a Bruker (Billerica, MA, USA) Avance III HD Cryo NMR spectrometer (700 MHz). Spectra were measured in C_6D_6 and referenced against solvent signals (^{1}H-NMR, residual proton signal: δ = 7.16 ppm; ^{13}C-NMR: δ = 128.06 ppm).[3]

GC/MS analysis

GC/MS analyses were performed on an Agilent (Santa Clara, CA, USA) 7890B GC connected to a 5977A mass detector. A HP5-MS fused silica capillary column (30 m, 0.25 mm i. d., 0.50 μm film) was used for gas chromatographic separation. GC parameters were 1) inlet pressure: 77.1 kPa, He at 23.3 mL min^{-1}, 2) injection volume: 2 μL, 3) temperature program: 5 min at 50 °C, then increasing at 5 °C min^{-1} or 10 °C min^{-1} to 320 °C, 4) 60 s valve time, and 5) carrier gas: He at 1.2 mL min^{-1}. MS parameters were 1) source: 230 °C, 2) transfer line: 250 °C, 3) quadrupole: 150 °C and 4) electron energy: 70 eV.

Table S1. Isotopic labelling experiments for EI-MS fragmentation mechanism of **1**.

No.	substrates	enzymes	result shown in
1	DMAPP + (*E*)-(4-^{13}C, 4-^{2}H)IPP[4]	GGPPS[1] + SvS[1]	Figure S1
2	DMAPP + (*Z*)-(4-^{13}C, 4-^{2}H)IPP[4]	GGPPS + SvS	Figure S2
3	(*R*)-(1-^{13}C,1-^{2}H)IPP[2]	IDI[2] + GGPPS + SvS	Figure S3
4	(*S*)-(1-^{13}C,1-^{2}H)IPP[2]	IDI + GGPPS + SvS	Figure S4
5	FPP + (1-^{13}C)IPP[1]	GGPPS + SvS	Figure S5A
6	FPP + (2-^{13}C)IPP[5]	GGPPS + SvS	Figure S5B
7	FPP + (3-^{13}C)IPP[1]	GGPPS + SvS	Figure S5C
8	FPP + (4-^{13}C)IPP[1]	GGPPS + SvS	Figure S5D
9	(1-^{13}C)FPP[6] + IPP	GGPPS + SvS	Figure S5E
10	(2-^{13}C)FPP[6] + IPP	GGPPS + SvS	Figure S5F
11	(3-^{13}C)FPP[6] + IPP	GGPPS + SvS	Figure S5G
12	(4-^{13}C)FPP[6] + IPP	GGPPS + SvS	Figure S5H
13	(5-^{13}C)FPP[6] + IPP	GGPPS + SvS	Figure S5I
14	(6-^{13}C)FPP[6] + IPP	GGPPS + SvS	Figure S5J
15	(7-^{13}C)FPP[6] + IPP	GGPPS + SvS	Figure S5K
16	(8-^{13}C)FPP[6] + IPP	GGPPS + SvS	Figure S5L
17	(9-^{13}C)FPP[6] + IPP	GGPPS + SvS	Figure S5M
18	(10-^{13}C)FPP[6] + IPP	GGPPS + SvS	Figure S5N
19	(11-^{13}C)FPP[6] + IPP	GGPPS + SvS	Figure S5O
20	(12-^{13}C)FPP[6] + IPP	GGPPS + SvS	Figure S5P
21	(13-^{13}C)FPP[6] + IPP	GGPPS + SvS	Figure S5Q
22	(14-^{13}C)FPP[6] + IPP	GGPPS + SvS	Figure S5R
23	(15-^{13}C)FPP[6] + IPP	GGPPS + SvS	Figure S5S
24	FPP + (5-^{13}C)IPP[7]	GGPPS + SvS	Figure S5T

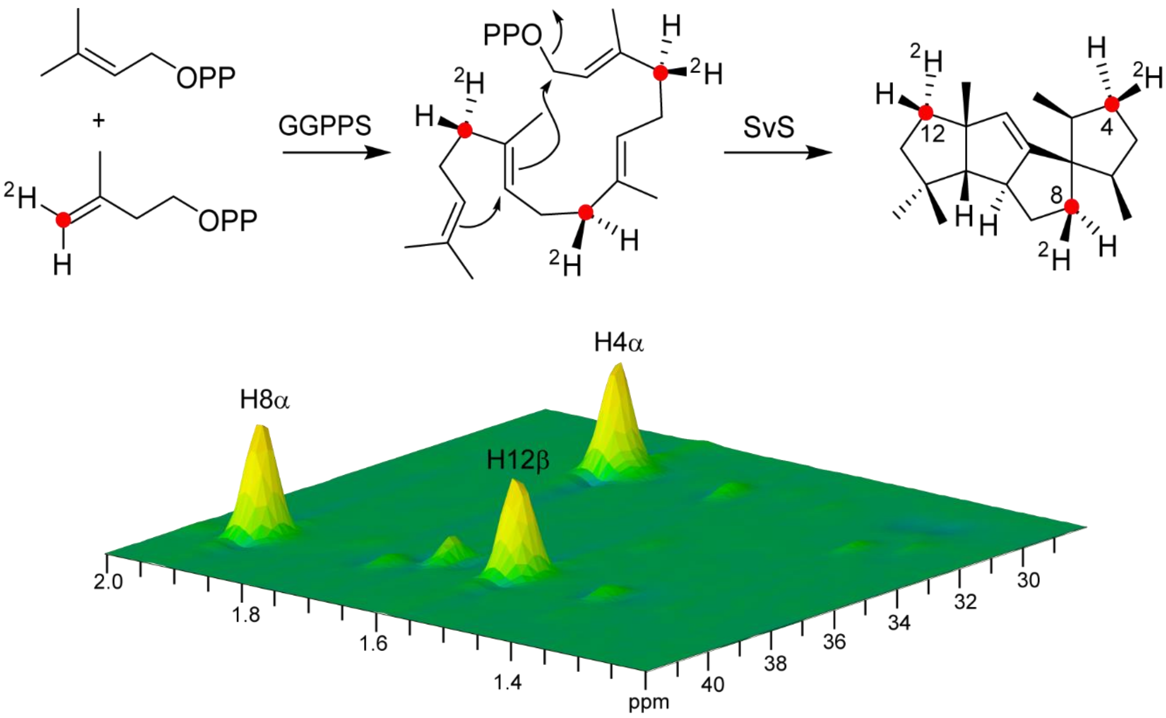

Figure S1. HSQC analysis of the product obtained by the enzymatic conversion of DMAPP and (*E*)-(4-^{13}C, 4-^{2}H)IPP with GGPPS and SvS. Red dots indicate ^{13}C-labellings.

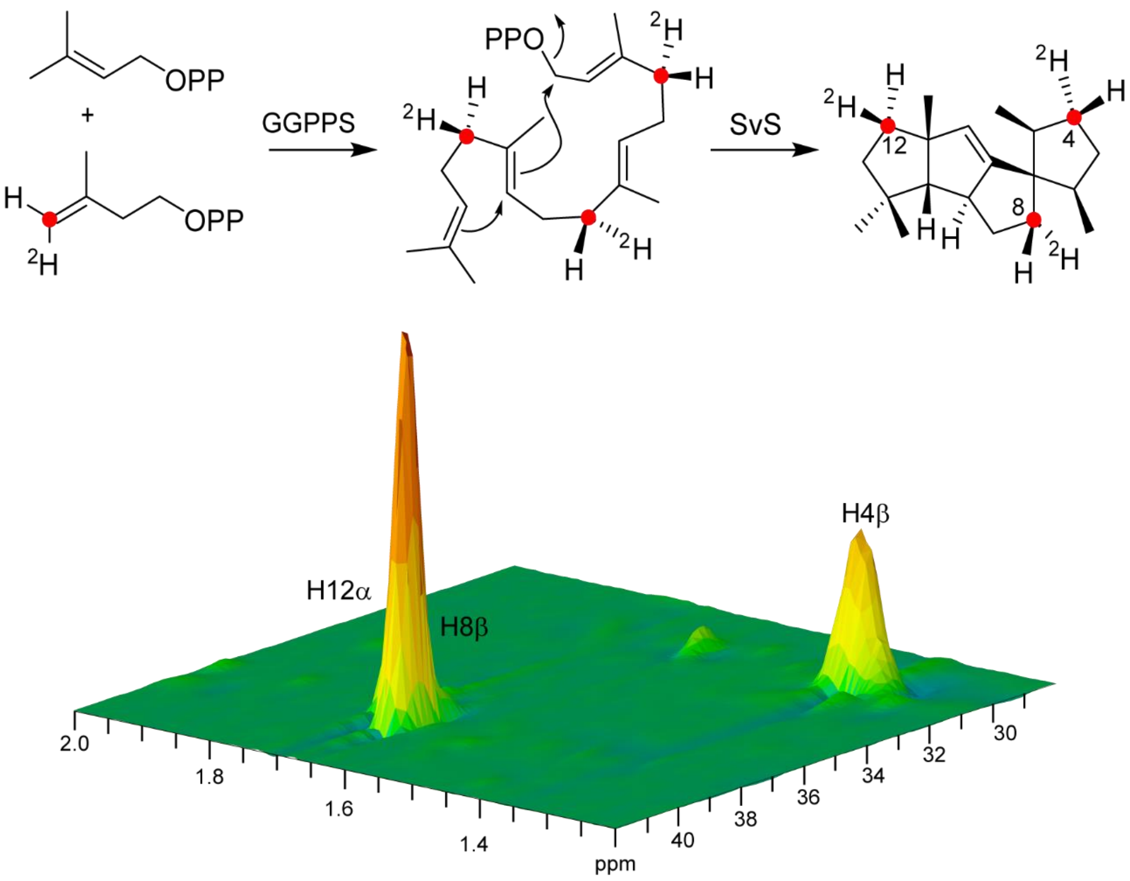

Figure S2. HSQC analysis of the product obtained by the enzymatic conversion of DMAPP and (*Z*)-(4-^{13}C, 4-^{2}H)IPP with GGPPS and SvS. Red dots indicate ^{13}C-labellings.

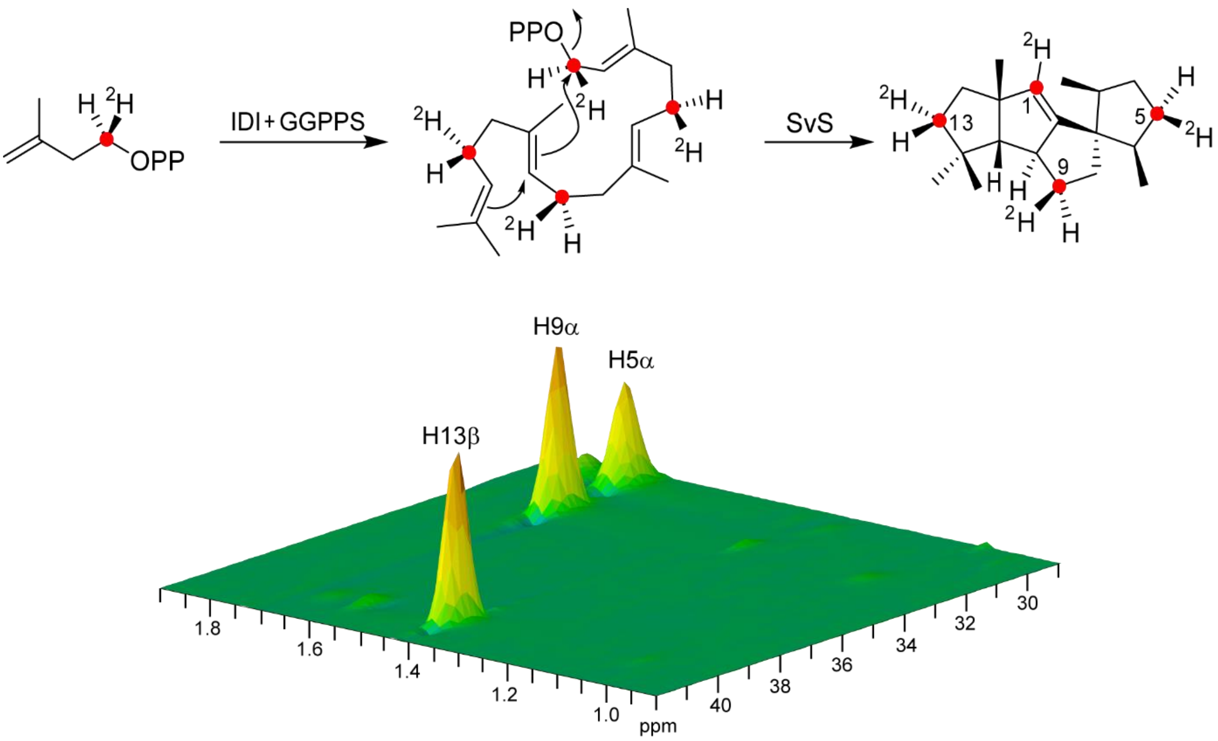

Figure S3. HSQC analysis of the product obtained by the enzymatic conversion of (*R*)-(1-^{13}C,1-^{2}H)IPP with IDI, GGPPS and SvS. Red dots indicate ^{13}C-labellings.

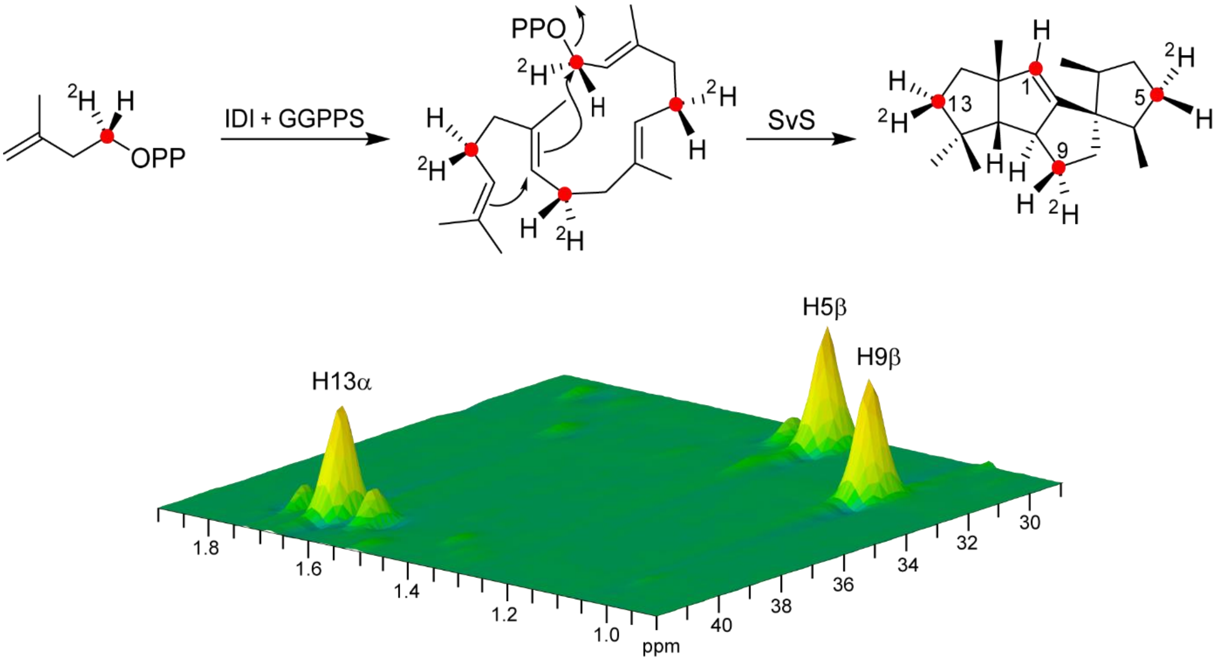

Figure S4. HSQC analysis of the product obtained by the enzymatic conversion of (*S*)-(1-^{13}C, 1-^{2}H)IPP with IDI, GGPPS and SvS. Red dots indicate ^{13}C-labellings.

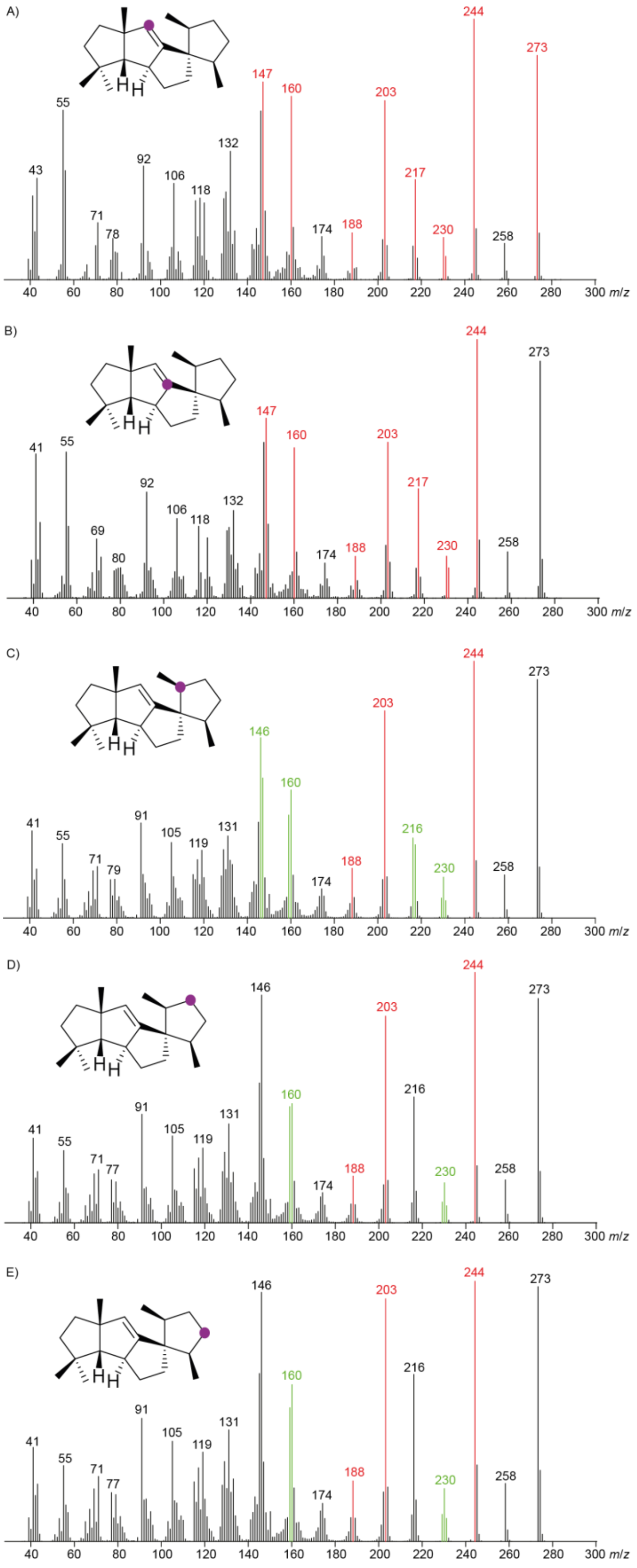

Figure S5. EI mass spectra of A) (1-^{13}C)-**1**, B) (2-^{13}C)-**1**, C) (3-^{13}C)-**1**, D) (4-^{13}C)-**1**, and E) (5-^{13}C)-**1**, obtained enzymatically with SvS from the corresponding isotopomers of (^{13}C)GGPP. Fragment ions that are clearly shifted compared to non-labelled 1 are shown in red, fragment ions that shift partially are shown in green (refers to colour code in Figure 1 of main text).

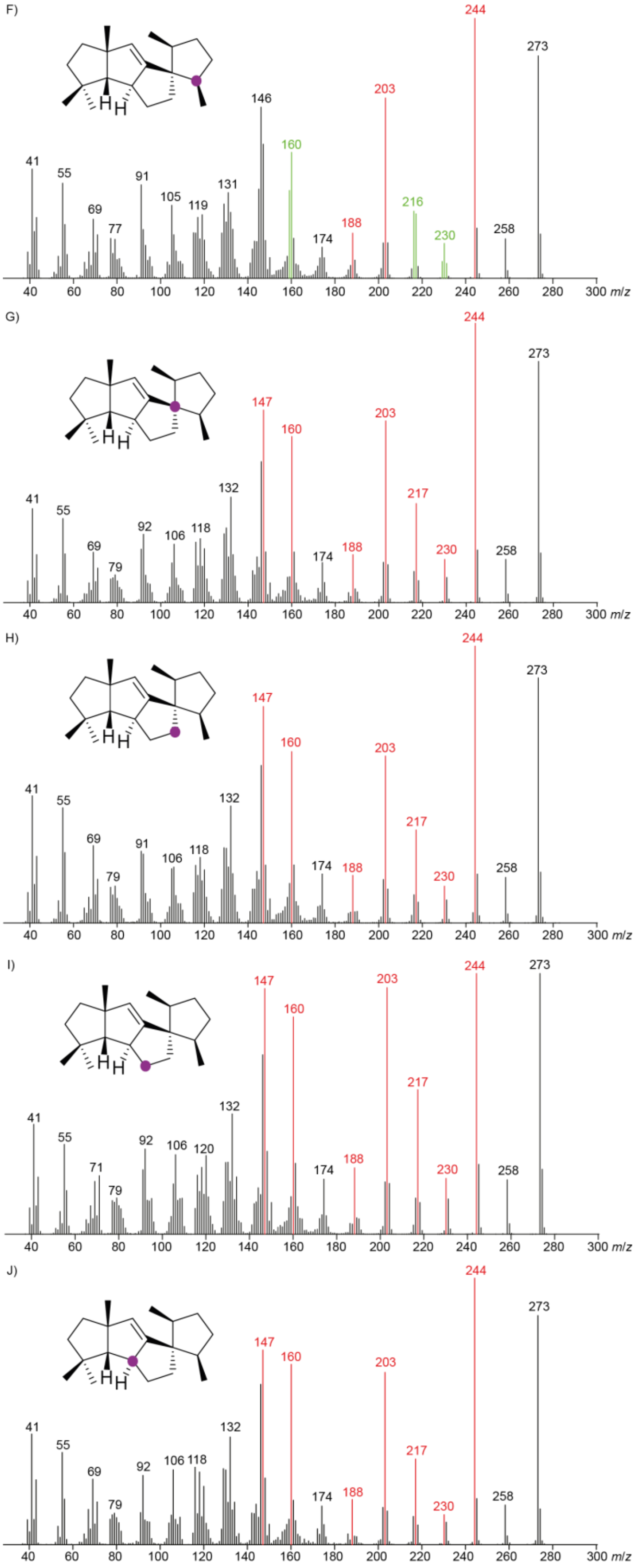

Figure S5. EI mass spectra of F) (6-^{13}C)-**1**, G) (7-^{13}C)-**1**, H) (8-^{13}C)-**1**, I) (9-^{13}C)-**1**, and J) (10-^{13}C)-**1**, obtained enzymatically with SvS from the corresponding isotopomers of (^{13}C)GGPP. Fragment ions that are clearly shifted compared to non-labelled 1 are shown in red, fragment ions that shift partially are shown in green (refers to colour code in Figure 1 of main text).

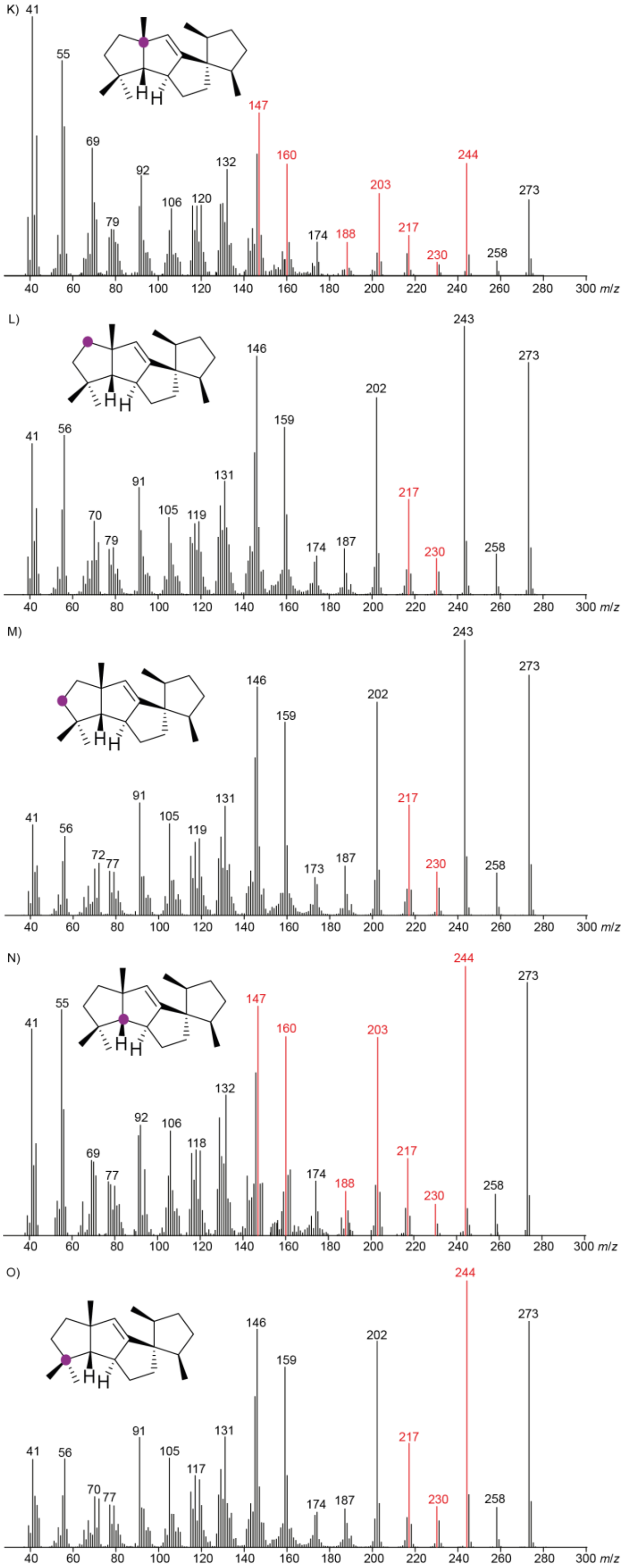

Figure S5. EI mass spectra of K) (11-^{13}C)-**1**, L) (12-^{13}C)-**1**, M) (13-^{13}C)-**1**, N) (14-^{13}C)-**1**, and O) (15-^{13}C)-**1**, obtained enzymatically with SvS from the corresponding isotopomers of (^{13}C)GGPP. Fragment ions that are clearly shifted compared to non-labelled 1 are shown in red, fragment ions that shift partially are shown in green (refers to colour code in Figure 1 of main text).

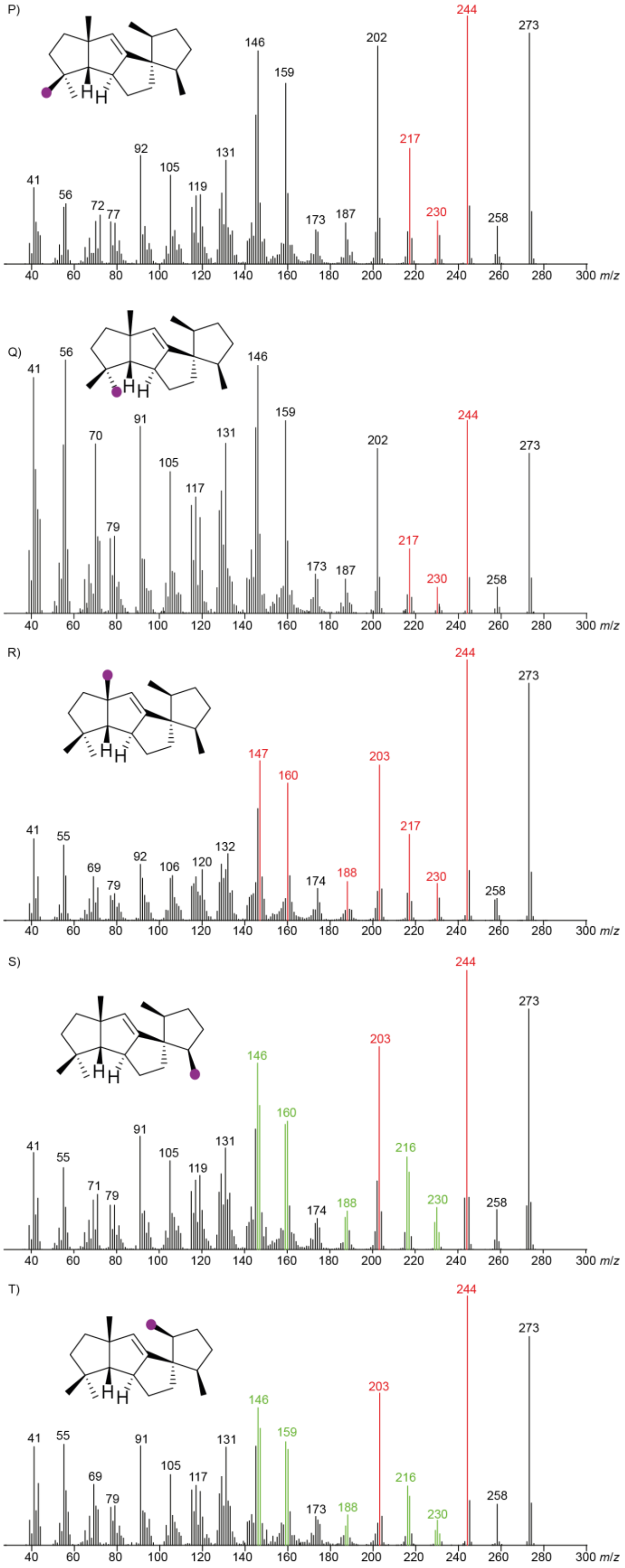

Figure S5. EI mass spectra of P) (16-^{13}C)-**1**, Q) (17-^{13}C)-**1**, R) (18-^{13}C)-**1**, S) (19-^{13}C)-**1**, and T) (20-^{13}C)-**1**, obtained enzymatically with SvS from the corresponding isotopomers of (^{13}C)GGPP. Fragment ions that are clearly shifted compared to non-labelled 1 are shown in red, fragment ions that shift partially are shown in green (refers to colour code in Figure 1 of main text).

References

[1] P. Rabe, J. Rinkel, E. Dolja, T. Schmitz, B. Nubbemeyer, T. H. Luu, J. S. Dickschat, *Angew. Chem. Int. Ed.* **2017**, *56*, 2776.

[2] J. Rinkel, J. S. Dickschat, *Org. Lett.* **2019**, *21*, 2426.

[3] G. R. Fulmer, A. J. M. Miller, N. H. Sherden, H. E. Gottlieb, A. Nudelman, B. M. Stoltz, J. E. Bercaw, K. I. Goldberg, *Organometallics* **2010**, *29*, 2176.

[4] L. Lauterbach, J. Rinkel, J. S. Dickschat, *Angew. Chem. Int. Ed.* **2018**, *57*, 8280.

[5] J. Rinkel, L. Lauterbach, J. S. Dickschat, *Angew. Chem. Int. Ed.* **2019**, *58*, 452.

[6] P. Rabe, L. Barra, J. Rinkel, R. Riclea, C. A. Citron, T. A. Klapschinski, A. Janusko, J. S. Dickschat, *Angew. Chem. Int. Ed.* **2015**, *54*, 13448.

[7] A. Hou, J. S. Dickschat, *Angew. Chem. Int. Ed.* **2020**, *59*, doi: 10.1002/anie.202010084.
